# Information Theoretic Methods for Variable Selection—A Review

**DOI:** 10.3390/e24081079

**Published:** 2022-08-04

**Authors:** Jan Mielniczuk

**Affiliations:** 1 Institute of Computer Science, Polish Academy of Sciences, Jana Kazimierza 5, 01-248 Warsaw, Poland; j.mielniczuk@ipipan.waw.pl; 2 Faculty of Mathematics and Information Science, Warsaw University of Technology, Koszykowa 75, 00-662 Warsaw, Poland

**Keywords:** conditional independence, interaction information, Möbius expansion, Markov blanket, feature selection

## Abstract

We review the principal information theoretic tools and their use for feature selection, with the main emphasis on classification problems with discrete features. Since it is known that empirical versions of conditional mutual information perform poorly for high-dimensional problems, we focus on various ways of constructing its counterparts and the properties and limitations of such methods. We present a unified way of constructing such measures based on truncation, or truncation and weighing, for the Möbius expansion of conditional mutual information. We also discuss the main approaches to feature selection which apply the introduced measures of conditional dependence, together with the ways of assessing the quality of the obtained vector of predictors. This involves discussion of recent results on asymptotic distributions of empirical counterparts of criteria, as well as advances in resampling.

## 1. Introduction

Conditional independence is one of the main concepts in statistics which plays a fundamental role in such areas as causal inference, dependence analysis, and graphical modelling. In this review, we discuss how measures of dependence considered in information theory are used in classification and regression problems to choose predictors which significantly influence the outcome. This is a vital application of the information theoretic approach to variable selection, also known as feature selection. Let us stress, however, that information-theoretic measures are frequently applied in many other problems in Machine Learning and Statistics, in contexts other than feature selection. Some representative examples include data visualisation (t-SNE, [1]), clustering [2], Independent Component Analysis (ICA [3], Chapter 15), Variational Inference ([4], Chapter 10), and Natural Language Modelling [5], among others.

In the times of the big data challenge, the problem of a choice of a small group of variables from the pool of all potential variables, all of which would accurately describe the changes in response, is gaining in importance. This is needed for better understanding of a studied phenomena, as well as for construction of tractable models for them. Moreover, feature selection is instrumental in avoiding curse of dimensionality problem when a prohibitive amount of data are required for adequate fitting of the model ([6], Chapter 2) and avoiding overfitting (ibid., Chapter 7). This is also important for prediction, as classifiers which avoid using inactive predictors are usually less variable. Feature selection is frequently applied because of cost considerations, especially when measuring some of the potentially useful predictors is costly or inconvenient.

Another competing direction of research with a similar aim is dimensionality reduction, in particular variable extraction, which transforms given variables to obtain a lean group of new predictors. Primary examples of such methods are Principal Components Regression and Partial Least Squares, see, e.g., [6], and more recent approaches based on neural networks [7].

Here, we focus on an important group of selectors, called *filters*, which, during the selection process, do not take into account the classifier (or regression estimator) which will incorporate selected variables. In contrast, *wrappers* select features to optimise performance of a specific classifier under consideration (see, e.g., methods which also yield ranking of features, such as [8,9,10]). The property that filters are model-agnostic makes them a universal tool which can be used for any classifier or regression estimator.

In this paper, we try to present critical and, unavoidably, partial assessment of the state of the art for this problem, focusing on results which can be formally proved, and showing motivation, advantages, and limitations of the presented solutions, including the most recent ones. Additionally, we show that the majority of information-based feature selection criteria can be viewed as truncated or truncated and weighted expansions of Möbius decomposition. This sheds new light on similarities and differences between the frequently employed criteria. Moreover, based on the recent results, asymptotic distributions of their plug-in empirical counterparts are discussed. Such results are needed to construct a test of conditional independence which control probability of false alarms. The recent advances in resampling yielding conditionally independent samples which provide alternative method to solve this problem are also discussed. By this, hopefully, a new insight is added to existing reviews (see e.g., [11,12,13,14]).

The paper is organised as follows. We first discuss in Section 2 the main information theoretic objects and their interplay, focusing on Möbius decomposition of conditional mutual information (CMI). In Section 3, we introduce and discuss a feature selection problem from the information theory perspective based on measures of dependence introduced in the previous section. The concept of Markov Blanket of a target variable, being the aim of feature selection as the minimal set of predictors containing the whole information about it, is investigated here. In Section 4, we study feature selection criteria related to CMI stressing that most of them are naturally related to Möbius decomposition. Moreover, this section introduces another group of selectors based on variational bounds of information measures. In the following Section 5, we discuss the interplay between various CMI-related measures. The next sections cover the approximate distributions of the introduced measures (Section 6) and an alternative method of assessing their distributions using resampling schemes (Section 7). These properties are vital for performance of conditional independence tests, which are building blocks of feature selection algorithms. In Section 8, Markov Blanket discovery algorithms are described. Section 9 discusses feature selection in continuous case, Section 10 covers related problem of interaction detection stressing how these can be incorporated into feature selection approach.

## 2. Conditional Mutual Information and Related Measures

We start with discussing properties and the role that conditional mutual information (CMI) plays in variable selection based on information theoretic concepts. We will focus on a discrete finite case, meaning that the considered variables take a finite number of discrete values. The continuous case is reviewed shortly in Section 9. In the following, *Y* will denote the class variable whereas *X* and *Z* (possibly multivariate) are features which will be used to predict *Y*. For basic information theoretic concepts we refer to [15,16].

### 2.1. Mutual Information MI

**Definition** **1** ([15], p. 46)**.** *The Mutual Information (MI) between Y and X is defined as*

(1) $$I\left(Y;X\right)=\sum_{x_{,}y}P\left(X=x,Y=y\right)\log\frac{P(X=x,Y=y)}{P(X=x)P(Y=y)}=H\left(Y\right)-H\left(Y|X\right),$$

*where $H\left(Y\right)=-\sum_{y}P\left(Y=y\right)\log P\left(Y=y\right)$ and $H\left(Y|X\right)=\sum_{x}P\left(X=x\right)H\left(Y|X=x\right)$ are the entropy and the conditional entropy, respectively.*

The second equality in (1) motivates the name *Information Gain* also used for MI. It also yields intuitive meaning for MI as the decrease in variability of *Y* is measured by its entropy when the information about another variable *X* is available. We remark that I(Y;X) is frequently denoted as MI(Y;X). Note that the semicolon in I(Y;X) determines between which variables dependence is considered: either between *Y* and *X* in the case of I(Y;X) or between *Y* and (X,Z) in the case of I(Y;X,Z). I(Y;X) evaluates how similar the joint distribution $P_{YX}$ of (Y,X) is to the product $P_{Y}⊗P_{X}$ of their marginal distributions. As $P_{Y}⊗P_{X}$ corresponds to independence of *X* and *Y*, I(Y;X) can be considered a measure of strength of dependence between *Y* and *X*. If follows from the definition that ([15], Section 2.3)

$$I\left(Y;X\right)=KL(P_{Y,X}||P_{Y}⊗P_{X}),$$

where *Kullback–Leibler (KL) divergence* between distributions $P_{W}$ and $P_{Z}$ is defined as

$$KL(P_{W}\left|\right|P_{Z})=\sum_{w}P\left(W=w\right)\log\left\{P\left(W=w\right)/P\left(Z=w\right)\right\}.$$

We note that KL divergence is closely related to the Maximum Likelihood (ML) method and popular feature selection method Akaike Information Criterion (AIC) is derived as the bias-corrected ML [17]. We also remark that Kullback–Leibler divergence can be replaced by other pseudo-distance between probability distributions resulting in a different measure of dependence.

It follows from the properties of KL divergence that I(Y;X) is non-negative and is equal to zero if, and only if, $P_{Y,X}=P_{Y}⊗P_{X}$, i.e., when *Y* and *X* are independent. Moreover, the definition (1) that Mutual Information is symmetric: I(Y;X)=I(X;Y). It is also easily seen that (see formula (2.450) in [15])

(2) I(Y;X)=H(Y)+H(X)−H(Y,X).

### 2.2. Conditional Mutual Information CMI

We denote by $P_{X|Z=z}$ conditional distribution of *X* given Z=z and define $I\left(Y;X|Z=z\right)=KL(P_{Y,X|Z=z}||P_{Y|Z=z}⊗P_{X|Z=z})$ as the strength of dependence between *Y* and *X* given Z=z. Thus, I(Y;X|Z=z)=0 means that *Y* and *X* are independent given that *Z* equals *z*. Now we define conditional mutual information.

**Definition** **2** ([15], p. 49)**.** *The conditional mutual information (CMI) is*

(3) $$\begin{array}{ccc} I(Y;X|Z) & = & E_{Z=z}I\left(Y;X|Z=z\right)\\ & = & \sum_{z}P\left(Z=z\right)\sum_{x,y}P\left(X=x,Y=y|Z=z\right)\log\frac{P(X=x,Y=y|Z=z)}{P(X=x|Z=z)P(Y=y|Z=z)}. \end{array}$$

Thus, the conditional mutual information is the mutual information of *Y* and *X* given Z=z averaged over the values of *Z*. It is measure of dependence between *Y* and *X* given the knowledge of *X*. From the properties of Kullback–Leibler divergence it follows that

I(Y;X|Z)=0⇔XandYareconditionallyindependentgivenZ.

This is a powerful property, not satisfied for other measures for dependence, such as partial correlation coefficient in the case of continuous random variables. The conditional independence of *X* and *Y* given *Z* will be denoted by X⊥⊥Y|Z and abbreviated to CI. We note that since I(Y;X|Z) is defined as a probabilistic average of I(Y;X|Z=z) over Z=z, it follows that

I(Y;X|Z)=0⇔I(Y;X|Z=z)=0foranyzinthesupportofZ.

Thus, formally, testing conditional independence of *X* and *Y* given *Z* is equivalent to testing unconditional dependence of *X* and *Y* on every stratum Z=z. This, however, does not make the problem an easy task, as sometimes claimed, since performing such tests simultaneously on each strata (global null), even when we have sufficient data to do it, would result in lack of control of the probability of false signals (multiple testing problem).

The above definitions can be naturally extended to the case of random vectors (i.e., when *X*, *Y*, and *Z* are multivariate) by using a multivariate probability mass functions instead of a univariate one. It is also easily seen that the following chain formula holds

(4) I(Y;X,Z)=I(Y;X)+I(Y;Z|X).

### 2.3. Interaction Information II

The 3-way interaction information II(Y;X,Z) of, possibly multivariate, X,Y, and *Z* plays also important role in feature selection.

**Definition** **3.** *The 3-way interaction information II(Y;X,Z) is defined as*

(5) II(Y;X;Z)=I(Y;X|Z)−I(Y;X)=I(Y;X,Z)−I(Y;X)−I(Y;Z).

The second equality, stemming from (4), neatly explains the idea of interaction information: we want to evaluate the synergistic effect (positive or negative) of both *X* and *Z* influencing *Y*, disregarding the sum of their individual effects. Thus, from the evaluated strength of *overall* dependence between *Y* and a pair (X,Z) measured by I(Y;X,Z), we subtract the strength of *individual* dependences of *Y* on *X* and *Y* on *Z* measured by I(Y;X) and I(Y;Z), respectively. Although it is not immediately clear from the definition, II(Y;X;Z) is actually a symmetric function of its arguments. However, it can be positive, negative, or 0. If it is positive, *X* and *Z* are said to be complementary wrt to *Y* or interacting positively in influencing *Y*. A smaller than 0 value of II indicates redundancy among features or their inhibition.

We define 2-way as II as

II(Y;X)=I(Y;X)andII(Y;X|Z)=I(Y;X|Z).

Definition (5) can be generalised in a recursive way to define *k*-way interaction information (see [18,19,20] for alternative equivalent definition). For any subset of indices $S=\{s_{1},s_{2},\ldots ,s_{\left|S\right|}\}$, $Z_{S}:=\left(Z_{s_{1}},Z_{s_{2}},\ldots ,Z_{s_{\left|S\right|}}\right)$ will stand for the sub-vector of $Z_{i}$s with indices in *S*. Abusing the notion, $Z\in Z_{S}$ will be mean that $Z\in \{Z_{s_{1}},Z_{s_{2}},\ldots ,Z_{s_{\left|S\right|}}\}$. Let S={1,…,|S|} and k=|S| be the cardinality of *S*. We define *k*-way interaction information $II(Z_{1};Z_{2};\ldots ;Z_{\left|S\right|})$ so that the chain formula holds.

**Definition** **4.** *k-way interaction information $II(Z_{1};Z_{2};\ldots ;Z_{\left|S\right|})$ is defined as*

(6) $$II\left(Z_{1};Z_{2};\ldots ;Z_{\left|S\right|}\right)=II\left(Z_{1};\ldots ;Z_{|S|-1}|Z_{\left|S\right|}\right)-II\left(Z_{1};\ldots ;Z_{|S|-1}\right),$$

*where, consistently with the definition of the conditional mutual information in (3), we define*

$$II\left(Z_{1};\ldots ;Z_{|S|-1}|Z_{\left|S\right|}\right)=\sum_{z_{\left|S\right|}}p\left(z_{\left|s\right|}\right)II\left(Z_{1};\ldots ;Z_{|S|-1}|Z_{\left|S\right|}=z_{\left|s\right|}\right).$$

Using expansions of MI and CMI in terms of entropies, the following formula for *k*-way II is obtained

(7) $$II\left(Z_{1};\ldots ;Z_{\left|S\right|}\right)=-\sum_{i=1}^{\left|S\right|}\sum_{T\subseteq S,|T|=i}{\left(-1\right)}^{\left|S|-|T\right|}H\left(Z_{T}\right),$$

where $Z_{T}=\left(Z_{t_{1}},\ldots ,Z_{t_{i}}\right)$ for $T=\{t_{1},\ldots ,t_{i}\}$. In particular, we have

$$II\left(Z_{1};Z_{2};Z_{3}\right)=H\left(Z_{1},Z_{2}\right)+H\left(Z_{2},Z_{3}\right)+H\left(Z_{1},Z_{3}\right)-H\left(Z_{1},Z_{2},Z_{3}\right)-H\left(Z_{1}\right)-H\left(Z_{2}\right)-H\left(Z_{3}\right).$$

Note that when, e.g., $Z_{3}=XOR\left(Z_{1},Z_{2}\right)=I\left\{Z_{1}\neq Z_{2}\right\}$ and $\left(Z_{1},Z_{2}\right)$ are independent copies of a random variable having Bernoulli distribution with p=1/2, we obtain $II(Z_{1};Z_{2};Z_{3})=\log2$, as in this case $I\left(Z_{3},Z_{2}|Z_{1}\right)=H\left(Z_{3}\right)$. In the following, so-called Möbius expansion (see, e.g., [20]) plays a crucial role.

**Theorem** **1.** *The conditional mutual information satisfies the equation*

(8) $$I\left(X;Y|Z_{S}\right)=I\left(Y;X|Z_{1},\ldots ,Z_{\left|S\right|}\right)=\sum_{i=0}^{\left|S\right|}\sum_{T\subseteq S,|T|=i}II\left(X;Y;Z_{t_{1}},\ldots ;Z_{t_{i}}\right),$$

*where $T=\{t_{1},\ldots ,t_{i}\}$ and, conversely,*

(9) $$II\left(X;Y;Z_{s_{1}},\ldots ,Z_{s_{\left|S\right|}}\right)=\sum_{i=0}^{\left|S\right|}\sum_{T\subseteq S,|T|=i}{\left(-1\right)}^{\left|S|-|T\right|}I\left(X;Y|Z_{T}\right).$$

We stress that the inner sum ranges over all *subsets* of *S*. For a description of set functions for which (8) and (9) are equivalent, see [20]. Note that we carefully distinguish between 3-way interaction information $II(X;Y;Z_{S})$ with multivariate $Z_{S}$ as the third component and $II(X;Y;Z_{1},\ldots ;Z_{\left|S\right|})$ being (|S|+2)-way interaction information between X,Y and all components $Z_{1},\ldots ,Z_{\left|S\right|}$. Equality (8) can be restated, in view of II(X;Y)=I(X;Y) and (6) as

(10) $$I\left(X;Y|Z_{S}\right)=I\left(X;Y\right)+\sum_{i=1}^{\left|S\right|}\sum_{T\subseteq S,|T|=i}\left[II\left(X;Z_{t_{1}};\ldots ;Z_{t_{i}}|Y\right)-II\left(X;Z_{t_{1}};\ldots ;Z_{t_{i}}\right)\right].$$

Finally, note that it follows from (9) that $II(X;Y;Z_{1},\ldots ;Z_{\left|S\right|})=0$ provided *X* and *Y* are conditionally independent given any subvector $Z_{T}$ of $Z_{S}$ including $Z_{\emptyset }$. As we shall see in Section 4.1, Möbius expansion (8) is a natural starting point to introduce CMI-related criteria for feature selection.

Let us indicate that for any classifier $\hat{Y}=Y\left(X\right)$ of class variable *Y* for *g* classes, its unconditional probability of error $P(Y\neq \hat{Y})$ can be related to conditional entropy H(Y|X) by means of Fano’s inequality ([15,21])

$$H\left(Y|X\right)\leq 1+P\left(Y\neq \hat{Y}\right)\log\left(g-1\right),$$

which, using I(X;Y)=H(Y)−H(Y|X), can be written as

$$P\left(Y\neq \hat{Y}\right)\geq \frac{H(Y)-I(X;Y)-1}{\log(g-1)}.$$

This shows that when I(Y;X) decreases, i.e., *Y* and *X* become less dependent, the lower bound on probability of error of any classifier increases.

## 3. Feature Selection

In this section we discuss an objective of feature selection, the concept of Markov Blanket and its properties, as well as a greedy search for active features.

### 3.1. General Considerations: Characterisations of Markow Blanket

Suppose now that we consider class variable *Y* and *p*-dimensional discrete vector $\left(X_{1},\ldots ,X_{p}\right)=:\left(Z_{1},\ldots ,Z_{p}\right)$ of all predictors available. Our aim is to describe *Y* using those features among $X_{1},\ldots ,X_{p}$ which jointly influence *Y*. Note that this is a different task than selecting features which individually affect *Y*. Namely, we want to find out which features can be discarded without loss of information about *Y* when the remaining features are taken into account. Thus, our aim is to check which features become redundant in the presence of other features. Moreover, we would like to investigate synergy between active features, that is a possible additional effect due to their simultaneous acting. Joint informativeness is, thus, crucial for feature selection. In this context, we define a minimal set of active predictors

**Definition** **5.** We define a minimal subset of active predictors as a minimal subset $S^{*}\subset \left\{1,\ldots ,p\right\}=:F$, such that

(11) $$I\left(Y;X_{S^{*}}\right)=\max_{T\subseteq F}I\left(Y;X_{T}\right)=I\left(Y;X_{F}\right),$$

where $X_{T}$ denotes the subvector of $\left(X_{1},\ldots ,X_{p}\right)$ with indices in set T. Minimality is meant in the set-theoretic sense i.e., $S^{*}$ is minimal if there is no proper subset W⊂S which satisfies (11).

Note that the second equality in (11) is due to chain formula as it follows that $I\left(Y;X_{T}\right)\leq I\left(Y;X_{T^{\prime }}\right)$ for $T\subseteq T^{\prime }$.

The problem of determining $S^{*}$ is a feature selection problem stated in information-theoretic setting, as $X_{S^{*}}$ may be considered as the minimal set describing adequately the overall dependence of *Y* on the available vector of predictors. The other possible way of defining a small subset of predictors which contain ’almost’ all information about target *Y* is, for a given value of hyperparameter γ>0, to consider the smallest subset $S_{\gamma }^{*}$, such that

$$I\left(Y;S_{\gamma }^{*}\right)\geq I\left(Y;X_{F}\right)-\gamma .$$

The other variant of the problem is its constrained version when a solution is sought for specific cardinality *k* of the feature set

(12) $$I\left(Y;X_{S^{*}}\right)=\max_{T\subseteq F,|T|=k}I\left(Y;X_{T}\right),$$

which involves |F|k evaluations of mutual information. We also refer to the related bottleneck problem in which information in *X* is compressed to a random variable *M* satisfying a given compression condition making it easy to transmit, which has maximal mutual information with *Y*, see, e.g., [22].

Note that since $I(Y;X_{S})$ is monotone in the second coordinate ($I\left(Y;X_{S}\right)\geq I(I\left(Y;X_{T}\right)$ for T⊆S), we have

I(Y;XF)≥1|F|k∑T⊆F,|T|=kI(Y;XT),

thus the RHS can be used as a criterion when looking for $S^{*}$ ([23]).

It is shown in [24] that when the distribution of $\left(X_{1},\ldots ,X_{p}\right)$ is determined by a subvector $X_{S^{*}}$ corresponding to a certain $S^{*}\subseteq F$ and is parametrised by $\tau^{*}$, finding a maximiser of $I(Y;X_{T})$ is a first step of maximising the log-likelihood L(T,τ) over *T* and τ under so called filter assumption stating that optimisations over *T* and over τ are independent. The problem of uniqueness of $S^{*}$ satisfying (11) is important and sometimes disregarded as a problem in feature selection.

**Remark** **1.** Note that although (11) is trivially satisfied for $S^{*}=F$ it does not mean that the minimal set in the sense of inclusion is uniquely defined. The obvious example is constructed by taking any Y,X, such that I(Y;X)>0 and letting $X_{1}=X$ and $X_{2}=f\left(X_{1}\right)$ where f is any 1−−1 transform which maps a set of values of X onto itself. In this case, $I\left(Y;X_{1}\right)=I\left(Y;X_{2}\right)=I\left(Y;X_{1},X_{2}\right)$, thus both subsets $\left\{X_{1}\right\}$ and $\left\{X_{2}\right\}$ satisfy (11) and are minimal. Moreover, note that $Y⊥\;\;\;⊥X_{2}|X_{1}$. However, uniqueness of $S^{*}$ satisfying (11) holds for the case of continuous X and Y binary under strict positiveness assumptions stating that density p(x) is positive almost everywhere with respect to Lebesgue measure and $P_{X}\left(p\left(Y=1|X\right)=1/2\right)=0$ (see [25], p. 97).

We discuss, now, properties of the set $S^{*}$. The first one is obtained by noticing that in view of the chain formula for $S^{*}$ defined in (11) we have $I(Y;X_{S^{*c}}|X_{S^{*}})=0$, where $T^{c}$ denotes complement of *T* in *F*. This is equivalent to stating that $S^{*}$ is so called *Markov Blanket* of *Y*.

**Definition** **6.** *Markov Blanket of target variable Y is the minimal subset MB(Y)⊆F, such that*

(13) $$X_{MB{\left(Y\right)}^{c}}⊥\;\;\;⊥Y|\;MB\left(Y\right),$$

*where $MB{\left(Y\right)}^{c}=\left\{1,\ldots ,p\right\}\backslash MB\left(Y\right)$ (Minimal set satisfying (13) is also called Markov boundary).*

Thus MB(Y) shields *Y* from the rest of predictors in the sense that they become irrelevant once MB(Y) is known. Again, MB(Y) does not need to be uniquely defined. In order to discuss properties of MB(Y) we introduce the concept of strong relevancy.

**Definition** **7.** *$X_{i}$ is strongly relevant feature provided*

$$I(Y;X_{i}|X_{F\backslash \{i\}})>0.$$

Note that the last property is equivalent to Y⫫Xi|XF\{i} and it can be restated as $P_{Y|X_{F}}\neq P_{Y|X_{F\backslash \{i\}}}$ [24]. Intuitively, strongly relevant features should be included in $S^{*}$ as, after exclusion of such features, the dependence of *Y* on $X_{F}$ is not adequately described. In the class of General Linear Models, under minimal conditions, features which are not strongly relevant are exactly those for which corresponding regression coefficient equals 0 (see [26], Proposition 2.2). The following facts concerning $S^{*}$ have been formally proved:

**Theorem** **2.** *Assume that Markov Blanket MB(Y) exists. Then, we have:*

*(i)*  $S^{*}$ coincides with Markov Blanket MB(Y);
*(ii)*  $S^{*}$ satisfies
(14) $$\forall T\subset F,\;\;\;T\subseteq F\backslash S^{*}\;\Leftrightarrow \;Y⊥\;\;\;⊥X_{T}|X_{S^{*}\backslash T};$$
*(iii)*  Every strongly relevant feature belongs to $S^{*}$.

The first statement is justified above. Part (ii) is due to [27] and indicates that all subsets of *F* are partitioned into two parts: the first consisting of sets *T* disjoint with $S^{*}$ for which $Y⊥\;\;\;⊥X_{T}|X_{S}^{*}$ holds and the remaining ones which are conditionally dependent with *Y* given $X_{S^{*}\backslash T}$.

We note that (iii) can be justified by the following simple reasoning: it is enough to show that if $j\notin S^{*}$ then $X_{j}$ is not strongly relevant. Indeed, we have

$$\begin{array}{ccc} & & I\left(Y;X_{S^{*}}\right)=I\left(Y;X_{F}\right)=I\left(Y;X_{F\backslash \{j\}}\right)+I\left(Y;X_{j}|X_{F\backslash \{j\}}\right)\\ & = & I\left(Y;X_{S^{*}}\right)+I\left(Y;X_{F\backslash \{\left\{j\right\}\cup S^{*}\}}|X_{S^{*}}\right)+I\left(Y;X_{j}|X_{F\backslash \{j\}}\right)\geq I\left(Y;X_{S^{*}}\right), \end{array}$$

where the second equality follows from the chain formula and the third from the fact that $j\notin S^{*}$ and the chain formula again. Thus, from the second equality it follows that $I(Y;X_{j}|X_{F\backslash \{j\}})=0$ whence $X_{j}$ is not strongly relevant. It is not true in general that MB(Y) consists only of strongly relevant features. Note that in the last example both $X_{1}$ and $X_{2}$ substituted for MB(Y) satisfy (13), but neither of them is strongly relevant as $Y⊥\;\;\;⊥X_{1}|X_{2}$ and $Y⊥\;\;\;⊥X_{2}|X_{1}$. In the case when *X* is continuous and *Y* is binary, this can be proved for strictly positive distributions defined in Remark 1 (cf. Theorem 10 in [28]).

Ref. [27] introduces a concept of *m*-Markov Blanket $S_{m}^{*}$ for m≤p for which equivalence in (14) is satisfied for any subset $T\subseteq F\backslash S_{m}^{*}$, such that |T|≤m. As for m=p the condition |T|≤m is vacuous, *p*-Markov Blanket of *Y* is MB(Y).

### 3.2. Greedy Feature Selection

In the view of (i) of Theorem 2, finding $S^{*}$ is equivalent to finding the minimal set satisfying

$$I(Y;X_{{S^{*}}^{c}}|X_{S^{*}})=0.$$

This is a difficult task when *p* is large as it would involve checking this condition for all $2^{p}$ subsets of {1,…,p}. The problem is frequently replaced by its greedy selection analogue: given *S* defined as the set of indices of features already selected, one determines

(15) $${\arg\max}_{j\in S^{c}}\left[I\left(X_{S\cup \{j\}};Y\right)-I\left(X_{S};Y\right)\right]={\arg\max}_{j\in S^{c}}I\left(X_{j};Y|X_{S}\right),$$

where the second equality follows from (4). The feature having the largest MI with *Y* is chosen first and the sequential search is stopped when for all $j\in S^{c}$ we have $I(X_{j};Y|X_{S})=0$. Note that, in view of chain equality, the maximised criterion equals

(16) $$I\left(X_{j};Y|X_{S}\right)=I\left(X_{j};Y\right)-I\left(X_{j};X_{S}\right)+I\left(X_{j};X_{S}|Y\right)=I\left(X_{j};Y\right)+II\left(X_{j};X_{S};Y\right),$$

where $II(X_{j},X_{S},Y)$ is *three-way* interaction information of $X_{j},X_{S}$, and *Y*. The first two terms of the first equality are called relevance and redundancy of $X_{j}$ wrt *Y* and $X_{S}$, respectively. Note that in the maximisation process of $I(X_{j};Y|X_{S})$ over $X_{j}$, redundancy of $X_{j}$ with respect to $X_{S}$ may be outweighed by the magnitude of conditional information which $X_{j}$ contains about $X_{S}$ within classes: $I(X_{j};X_{S}|Y)$. RHS of (16) involves $II(X_{j},X_{S},Y)$. In the next section, we show that $II(X_{j},X_{S},Y)$ is frequently replaced by algebraic expressions involving $II(X_{j};X_{t_{1}};\ldots ;X_{t_{k}};Y)$ where *k* does not exceed 2. This is motivated by (8) and allows for easier estimation of the expression.

## 4. Feature Selection Criteria Related to CMI

### 4.1. Criteria Based on Möbius Expansion

As estimation of conditional quantities, such as $I(Y;X|X_{S})$ and its effective use for detecting conditional dependence in case when $X_{S}$ is high-dimensional, requires large sample sizes (see, e.g., [29], Section 3.2), the common approach is to modify expressions, such as RHS of (10) to define analogues of CMI. Consider two approaches to achieve this aim. The first is based on truncation of the sum in (10) at a certain order, the second consists of weighing the corresponding terms and truncating them. In both cases, conditioning by random variable $X_{S}$ is replaced with conditioning by low-dimensional sub-vectors of these vector, which drastically reduces the need for large sample sizes. This makes them easier to estimate and analyse. As a motivational example, consider the situation when $X_{S}$ consists of |S|=10 binary coordinates, uniformly distributed on {0,1} and the sample size n=1000. Then, we expect around $1000/2^{10}\approx 1$ observation for each value $X_{S}=x_{S}$ of the conditioning variable $X_{S}$ in $I(Y;X|X_{S})$ whereas the respective number is around 500 for a single conditioning variable $X_{i}$ appearing in the definition of, e.g., JMI in (21) below. The problem that low dimensionality of conditioning set facilitates estimation is recognised (see, e.g., [29]).

More specifically, we will consider below the following criteria based on truncation:

- Mutual Information Maximisation criterion MIM;
- Conditional Infomax Feature Selection criterion CIFE of order two and three.

Additionally, the following criteria based on truncation and weighing of Möbius expansion will be considered:

- Generalised Information Criterion GIC;
- Joint Mutual Information criterion JMI of order two and three;
- Mutual Information Feature Selection criterion MIFS;
- Minimum-Redundancy Maximum-Relevance criterion mRMR.

The simplest approach is to consider only the first term of expansion (10) leading to *Mutual Information Maximisation* criterion MIM(X):=I(X;Y) (cf. [30]). This completely ignores dependence structure between *Y* and $X_{S}$, thus taking into account only feature relevance and disregarding its redundancy. However, it is useful and frequently applied for preliminary screening of predictors which are than subjected to more precise scrutiny. Actually, the name ’filters’ is frequently used for exactly such criteria when MI is replaced by dependence measure between *Y* and *X* of user’s choice.

Consideration of the first two summands of expansion (10) leads to *Conditional Infomax Feature Selection* (CIFE, [31], see also [24])

(17) $$CIFE\left(X\right)=CIFE\left(X,Y|X_{S}\right)=I\left(X;Y\right)+\sum_{i\in S}II\left(Y;X;X_{i}\right),$$

which is also called Short Expansion of CMI of order 2 (SECMI2) in [29].

We stress that, in the definition of CIFE and in definitions of the following criteria, the argument of the criterion is $X:=X_{i}$ for $i\in S^{c}$ which is the variable over which maximisation is performed.

Analogously, taking into account the first three summands in (8) yield [32]

(18) $$CIFE3\left(X\right)=I\left(X;Y\right)+\sum_{i\in S}II\left(Y;X;X_{i}\right)+\sum_{i<j,i,j\in S}II\left(Y;X;X_{i};X_{j}\right),$$

which is also called, stressing its relation to CMI, SECMI3. Thus, for |S|≤2, CIFE3 in greedy selection rule yields the same results as (15).

Incorporating weights into (10) leads to the following definition of *Generalised Information Criterion* (GIC): for any $\beta ,\gamma \in R^{\left|S\right|}$ we define

(19) $$I_{\beta ,\gamma }\left(X\right)=I\left(X;Y\right)+\sum_{k=1}^{\left|S\right|}\sum_{T=\{t_{1},\ldots ,t_{k}\}\subseteq S}\left[\gamma \left(k\right)II\left(X,X_{t_{1}};\ldots ;X_{t_{k}}\right)|Y\right)-\beta \left(k\right)II\left(X,X_{t_{1}};\ldots ;X_{t_{k}}\right)]),$$

where β=(β(1),…,β(k)) and γ=(γ(1),…,γ(k)). Usually β(l)=γ(l)=0 for $l\geq l_{0}$, where $l_{0}$ is a predefined small integer. Letting β(l)=γ(l)=0 for l≥2 one obtains criteria introduced in [24] parametrised by β and γ, where, abusing the notion slightly, β:=β(1) and γ:=γ(1):

(20) $$J_{\beta ,\gamma }\left(X\right)=I\left(X;Y\right)+\gamma \sum_{i=1}^{\left|S\right|}I\left(X;X_{i}|Y\right)-\beta \sum_{i=1}^{\left|S\right|}I\left(X;X_{i}\right).$$

For $\beta =\gamma ={\left|S\right|}^{-1}$*Joint Mutual Information* criterion (JMI) is obtained [33]:

(21) $$JMI\left(X\right)=I\left(Y;X\right)+\frac{1}{\left|S\right|}\sum_{i=1}^{\left|S\right|}\left(I\left(X;X_{i}|Y\right)-I\left(X,Z_{i}\right)\right)=I\left(Y;X\right)+\frac{1}{\left|S\right|}\sum_{k=1}^{\left|S\right|}II\left(Y;X;X_{i}\right).$$

Note that, in comparison to SECMI2 in (17), interaction terms are down-weighted by a factor ${\left|S\right|}^{-1}$.

Remembering that II is symmetric and writing $II\left(Y;X,Z_{i}\right)=I\left(Y;X|Z_{i}\right)-I\left(Y;X\right)$ one arrives at an useful form of JMI

(22) $$JMI\left(X\right)=\frac{1}{\left|S\right|}\sum_{i=1}^{\left|S\right|}I\left(Y;X|X_{i}\right),$$

which, in particular, shows that JMI=0 is equivalent to $Y⊥\;\;\;⊥X|X_{i}$ for any i∈S.

**Remark** **2.** *Note that JMI(X) is always non-negative, but it is not the case for CIFE(X) (and neither for CIFE3(X)). Indeed, taking arbitrary X such that $I(Y,X_{1})>0$ and letting $X_{2}=\cdots =X_{p}=X_{1}$ it is easy to check that w have for for p>2:*

$$CIFE\left(X_{1},Y|X_{2},\ldots ,X_{p}\right)=I\left(X_{1};Y\right)+\sum_{j=2}^{p}\left(I\left(X_{j};Y|X_{1}\right)-I\left(X_{j};Y\right)\right)=-\left(p-2\right)I\left(X_{1};Y\right)<0$$

*as $I(X_{j};Y|X_{1})=0$ (cf. also [34]).*

Letting γ=0 and β∈[0,1] one obtains *Mutual Information Feature Selection* (MIFS) criterion [35]

$$MIFS_{\beta }\left(X\right)=I\left(X;Y\right)-\beta \sum_{k=1}^{\left|S\right|}I\left(X;X_{i}\right),$$

with a special case β=1/|S| called *Minimum-Redundancy Maximum-Relevance* criterion (mRMR) introduced in [36]. A normalised version of the MIFS criterion was considered in [37].

In [38], 3-way JMI has been introduced by starting from the equality

$$\sum_{\{i,j\}\subseteq S}I\left(Y;X,X_{i},X_{j}\right)=\sum_{\{i,j\}\subseteq S}I\left(Y;X_{i},X_{j}\right)+\sum_{\{i,j\}\subseteq S}I\left(Y;X|X_{i},X_{j}\right).$$

Note that by dropping the first sum on RHS which does not depend on *X* (as the introduced criteria will be used to choose *X*) and scaling the second term by 2/(|S|(|S|−1) one obtains in view of (8)

(23) $$\begin{array}{ccc} JMI3(X) & = & \frac{2}{S|(|S|-1)}\sum_{\{i,j\}\subseteq S}I\left(Y;X|X_{i},X_{j}\right)\\ & = & I\left(Y;X\right)+\frac{2}{\left|S\right|}\sum_{i\in S}II\left(Y;X;X_{i}\right)+\frac{2}{\left|S|(|S|-1\right)}\sum_{i<j}II\left(Y;X;X_{i};X_{j}\right). \end{array}$$

This is generalised information criterion (20) with β(1)=γ(1)=2/|S| and β(1)=γ(1)=2/(|S|(|S|−1) and all other coefficients equal to 0.

The important criterion *Conditional Mutual Information Maximisation* CMIM using a different approach consisting of considering non-linear function of $I(Y;X|Z_{i}),i=1,\ldots ,|S|$ has been proposed in [39]

(24) $$CMIM\left(X\right)=\min_{j\in S}I\left(Y;X|X_{j}\right)=I\left(Y;X\right)-\max_{j\in S}\left[I\left(X;X_{j}\right)-I\left(X;X_{j}|Y\right)\right),$$

which should be maximised over $X:=X_{i}$ for $i\in S^{c}$. Thus, we look for the best surrogate of $X_{i}$ among already chosen variables and the candidate having the worst the best surrogate of *X* is chosen. Generalisation of the rule based on CMIM is considered in [40].

### 4.2. Variational Approach

The other promising approach to approximate MI and CMI is based on construction of variational lower bounds of these quantities. The bounds obtained are then used as selection criteria. We discuss two approaches which are similar in nature. The first one is based on Donsker–Varadhan inequality which states that [41]

(25) $$I\left(Y;X\right)\geq \underset{f\in F}{\sup}\left[E_{P_{X,Y}}f\left(X,Y\right)-\log E_{P_{X}⊗P_{Y}}e^{f(X,Y)}\right],$$

and inequality becomes equality when family F contains the logarithm of the ratio of densities of $P_{X,Y}$ and $P_{X}⊗P_{Y}$, namely $h\left(x,y\right)=\log(f_{XY}\left(x,y\right)/f_{X}\left(x\right)f_{Y}\left(y\right))$. Indeed, note that by plugging h(x,y)+C into the expression on the RHS of (25) we obtain equality. Estimation of I(X;Y) based on the Donsker–Varadhan formula has been proposed in [42] where neural network is applied for F resulting in MINE method. The approach has been further pursued in [43,44]. Other lower bounds, such as Nguyen–Wainwright–Jordan bound [45] can also be used. Note that in order to evaluate the expected value under independence in (25) one uses permutations of the original sample which consists in permuting *X* values while keeping the values of *Y* at their original places (see Section 7). For feature selection, one can either directly evaluate $I(Y;X_{S\cup \{j\}})$ or $I(Y;X_{j}|X_{S})$. For the second approach resampling methods which would ensure conditional independence for generated data are needed. Note that the efficiency of such methods depends on flexibility of function class F over which the bound in (25) is optimised. The empirical evidence suggest that relatively simple neural nets are sufficiently flexible to yield satisfactory estimators of I(Y;X). They are also preferable, as large networks will increase the variability of the estimators.

The second bound, similar in flavour to (25), is due to [46]. It is noticed there that for arbitrary density q(x,y) we have

$$\begin{array}{ccc} I(Y;X) & = & H\left(Y\right)-H\left(Y|X\right)=H\left(Y\right)+E_{P_{X}}E_{P_{Y|X}}\log p\left(Y|X\right)\\ & \geq & H\left(Y\right)+E_{P_{X}}E_{P_{Y|X}}\log q\left(Y|X\right)=E_{P_{X,Y}}\log\frac{q(X|Y)}{p(Y)}, \end{array}$$

where the inequality is due to D(p(Y|x)||q(Y|x))≥0. Assuming that q(y)=p(y) in view of Bayes theorem it is now sufficient to specify marginal density q(x|y) to obtain a lower bound on I(Y;X). In [46] q(x|y) are considered which either satisfy naive Bayes assumption or more general assumption that $q(x_{t}|y,x_{1},\ldots ,x_{t-1})$ is geometric mean of $q(x_{t}|y,x_{i})$ for i=1,…,t−1. Other proposals, using specific tree-representation for q(x|y) are possible (see, e.g., [47]).

## 5. Interplay between CMI and CMI-Related Criteria

The following result states conditions under which CMI and one of the introduced criteria coincide. As before, *X* denotes a feature from $X_{F\backslash S}$ considered as a candidate to be added to $\left\{X_{s},s\in S\right\}$.

**Theorem** **3.**

*(i)*  *Assume that all features are conditionally independent given class (naive Bayes assumption): and features in S are conditionally independent given any not chosen feature (i.e., belonging to $S^{c}$). Then, for $X\in X_{F\backslash S}$, $I(Y;X|X_{S})$ differs from $MIFS_{1}\left(X\right)=I\left(Y;X\right)-\sum_{i\in S}I\left(X;X_{i}\right)$ by a factor which does not depend on X.*
*(ii)*  *Assume that all k-way interaction informations $II(Y;X;X_{t_{1}};\ldots ;X_{t_{l}})=0$ for k>3 (l>1). Then, $I\left(Y;X|X_{S}\right)=CIFE\left(Y,X|X_{S}\right)$ for any $X\in X_{F\backslash S}$.*
*(iii)*  *Ref. [24] If $X_{i},i\in S$ are conditionally independent given X, for $X\in X_{F\backslash S}$ and additionally they are conditionally independent given X and Y, then $I(Y;X|X_{S})$ differs from $CIFE(Y,X|X_{S})$ by a factor which does not depend on X.*
*(iv)*  *Ref. [48] Assume that for any i∈S we have $X⊥\;\;\;⊥X_{S\backslash \{i\}}|X_{i}$ and additionally $X⊥\;\;\;⊥X_{S\backslash \{i\}}|X_{i},Y$. Then, $I\left(Y;X|X_{S}\right)=JMI\left(Y;X|X_{S}\right)$.*

For completeness, the proof is included in the Appendix A. Note that the conditions imposed by (i) are very stringent. There are no known probabilistic vectors satisfying $I(Y;X;X_{t_{1}},\ldots ;X_{t_{l}})=0$ for l>1 apart from simple situations, such as $X_{S}⊥\;\;\;⊥\left(X,Y\right)$, which obviously does not hold when $X_{S}$ is the set of chosen predictors.

We remark that under conditions of (i) $MIFS_{1}\left(X\right)=CIFE\left(X\right)$, as due to the naive Bayes assumption $\sum_{i\in S}I\left(X_{i};X|Y\right)=0$. Additionally, it follows from (21) that JMI(X)=mRMR(X) provided that the naive Bayes condition holds, thus in order to have JMI(X)=CMI(X)=mRMR(X) under rather strong assumptions of (iv), we have to assume additionally naive Bayes condition. This indicates that the last equality can hold only under restrictive conditions and is not true in general (see [24], Section 4.1).

Assumptions in (iii) and (iv) are not compatible as they lead to different forms of criteria equivalent to CMI criterion. It is argued in [46] that the only plausible graph representation of probability structure for which conditions of (iii) is graph with edges $E=\left\{\left(Y,X\right)\right\}\cup {\left\{\left(X,X_{i}\right)\right\}}_{i\in S}\}$. In this case, due to data-processing inequality, we have $I\left(Y;X\right)\geq I\left(Y;X_{S}\right)$. This means, however, that *X* should have been chosen *before* any features from *S*.

Additional results of similar flavours to Theorem 3 are discussed in [38,49].

It follows from preceding discussion that criteria introduced in Section 4.1 are formally introduced by truncation (or truncation and weighing) of terms in Möbius expansion. Their analytical properties concerning how well they approximate CMI have yet to be established.

## 6. Asymptotic Distributions of Information-Theoretic Measures

Sequential feature selection for predicting target variable *Y* typically involves checking whether a new candidate feature *X* is a valuable addition to the set of already chosen features $X_{S}$. This is usually based on testing whether null hypothesis $H_{0}:X⊥\;\;\;⊥Y|X_{S}$ holds and its rejection is interpreted as an indication that *X* carries an additional information about *Y* to that provided by $X_{S}$. To this end, $\hat{I}\left(Y;X|X_{S}\right)$ or its modified versions are used. The usual strategy following statistical testing approach is to derive asymptotic distribution of $\hat{I}\left(Y;X|X_{S}\right)$ or its modifications described in Section 4 under the null. This distribution is used as a benchmark for which value of the statistic for the sample under consideration is compared to obtain the asymptotic *p*-value of the test. In the following, we describe the asymptotic distribution of $\hat{CMI}$ and $\hat{CMI}$-related criteria under $H_{0}$. A competing approach to approximate the distribution of the considered statistic under $H_{0}$ based on resampling is described in Section 7.

### 6.1. Asymptotic Distribution of $\hat{CMI}$

We assume that $X,Y,X_{S}$ take I,J,K possible values, respectively. As $X_{S}$ is a |S|-dimensional vector, *K* is the number of all possible combinations of values of its coordinates. We let $p\left(x,y,x_{S}\right)=P\left(X=x,Y=y,X_{S}=x_{S}\right)$ and consider the case when all probabilities $p(x,y,x_{S})$ are positive. It is assumed throughout that the estimation of $I(Y;X|X_{S})$ and related quantities is based on *n* independent identically distributed (iid) samples from the distribution of $\left(X,Y,X_{S}\right)$. Construction of estimators of CMI relies on plugging-in frequencies in place of unknown probabilities, e.g.,

(26) $$\hat{I}\left(Y;X|X_{S}\right)=\sum_{x,y,x_{S}}\hat{p}\left(x,y,x_{S}\right)\log\frac{\hat{p}\left(x,y|x_{S}\right)}{\hat{p}\left(x|x_{S}\right)\hat{p}\left(y|x_{S}\right)}=\sum_{x,y,x_{S}}\hat{p}\left(x,y,x_{S}\right)\log\frac{\hat{p}\left(x,y,x_{S}\right)\hat{p}\left(x_{S}\right)}{\hat{p}\left(x,x_{S}\right)\hat{p}\left(y,x_{S}\right)},$$

where $\hat{p}\left(x,y,x_{S}\right)=n\left(x,y,x_{S}\right)/n$ and $n(x,y,x_{S})$ is a number of samples equal to $\left(x,y,x_{S}\right)$. We will consider only frequencies as estimators of discrete probabilities. Other estimators exist, e.g., regularised versions of sample frequencies, for which regularisation reflects a level of departure from conditional independence, see [38,50]. The following known result is frequently used in dependence analysis (compare [51], see also [52]).

**Theorem** **4.**

*(i)*  *Assume that $I(Y;X|X_{S})\neq 0$. Then, we have*
(27) $$n^{1/2}\left(\hat{I}\left(Y;X|X_{S}\right)-I\left(Y;X|X_{S}\right)\right)\overset{d}{\to }N\left(0,\sigma_{\hat{CMI}}^{2}\right),$$
  *where*
$$\sigma_{\hat{CMI}}^{2}=\sum_{x,y,x_{S}}p\left(x,y,x_{S}\right)\log^{2}\frac{p\left(x,y,x_{S}\right)p\left(x_{S}\right)}{p\left(x,x_{S}\right)p\left(y,x_{S}\right)}-I^{2}\left(X,Y|X_{S}\right)=\mathrm{Var}\left(\log\frac{p\left(X,Y,X_{S}\right)p\left(X_{S}\right)}{p\left(X,X_{S}\right)p\left(Y,X_{S}\right)}\right)$$
*and $\sigma_{\hat{CMI}}^{2}>0$.*
*(ii)*  *Assume that $I(Y;X|X_{S})=0$. Then,*
(28) $$2n\hat{I}\left(Y;X|X_{S}\right)\overset{d}{\to }\chi_{d}^{2},$$
  *where d=(I−1)(J−1)K.*

The frequently applied test of conditional independence $X⊥\;\;\;⊥Y|X_{S}$ is based on the above useful fact (ii) that under independence asymptotic distribution $\hat{I}\left(Y;X|X_{S}\right)$ does not depend on the distribution of $\left(X,Y,X_{S}\right)$ and is chi-square with the known number of degrees of freedom. On the other hand, when the conditional independence does not hold, the limiting distribution of $\hat{I}\left(Y;X|Z\right)-I\left(Y;X|X_{S}\right)$ is normal with the variance depending on the underlying probability distribution $P_{XYZ}$. We stress that speeds of convergence of $\hat{I}\left(Y;X|X_{S}\right)$ to $I(Y;X|X_{S})$ are different in both cases: they equal $n^{-1}$ in the first case and $n^{-1/2}$ in the second.

The test based on CMI is a popular tool in dependence analysis, in particular for Markov Blanket discovery (see Section 8). It has different names among which $G^{2}$ test is the most popular (see, e.g., [53]). Additionally, in the literature $X^{2}$ denotes the second order approximation of $\hat{CMI}$, which turns out to be the conditional chi-square test and has the same asymptotic distribution as $\hat{I}\left(Y;X|X_{S}\right)$.

### 6.2. Asymptotic Distribution of Modified Criteria

Asymptotic distribution of the modified criteria related to $\hat{CMI}$ can be also derived. Let $\hat{J^{\beta ,\gamma }}\left(X,Y|Z\right)$ be a plug in-version of $J^{\beta ,\gamma }$ defined in (20). Moreover, let $p=(p\left(x,y,x_{s}\right))$ be a vector of probabilities of dimension M=I×J×K, $\hat{p}$ corresponding vector of fractions and $f:{\left[0,1\right]}^{M}\to R$ be a function which represents $J^{\beta ,\gamma }$ as a function of *p*, i.e., $J^{\beta ,\gamma }=f\left(p\right)$. For example for JMI criterion (see (22)) the corresponding function is defined as

$$f_{JMI}\left(p\right):=\sum_{x,y,x_{S}}p\left(x,y,x_{S}\right)\frac{1}{\left|S\right|}\sum_{s\in S}\ln\frac{p\left(x,y,x_{s}\right)p\left(x_{s}\right)}{p\left(x,x_{s}\right)p\left(y,x_{s}\right)},$$

where $x_{s}$ denotes coordinate of $x_{S}$. We state here the result on asymptotic distribution of $\hat{J^{\beta ,\gamma }}\left(X,Y|X_{S}\right)$ proved in [29]. Let $\Sigma_{x,y,x_{S}}^{x{,}^{\prime }y^{\prime },x_{S}^{\prime }}$ denote an element of matrix Σ with row index $x,y,x_{S}$ and column index $x^{\prime },y^{\prime },x_{S}^{\prime }$.

**Theorem** **5.**

*(i)*  *We have*
(29) $$n^{1/2}\left(\hat{J^{\beta ,\gamma }}\left(X,Y|X_{S}\right)-J^{\beta ,\gamma }\left(X,Y|X_{S}\right)\right)\overset{d}{\to }N\left(0,\sigma_{\hat{J}}^{2}\right),$$
  *where $\sigma_{\hat{J}}^{2}=Df{\left(p\right)}^{T}\Sigma Df\left(p\right)=\mathrm{Var}\left(Df{\left(p\right)}^{T}\hat{p}\right)$ and $\Sigma =n\Sigma_{\hat{p}}$ is a matrix consisting of elements $\Sigma_{x,y,x_{S}}^{x^{\prime },y^{\prime },x_{S}^{\prime }}=p(x^{\prime },y^{\prime },x_{S}^{\prime })\left(I\left(x=x^{\prime },y=y^{\prime },x_{s}=x_{S}^{\prime }\right)-p\left(x,y,x_{S}\right)\right)/n$.*
*(ii)*  *If $\sigma_{\hat{J}}^{2}=0$ then*
(30) $$2n\left(\hat{J^{\beta ,\gamma }}\left(X,Y|X_{S}\right)-J^{\beta ,\gamma }\left(X,Y|X_{S}\right)\right)\overset{d}{\to }V^{T}HV,$$
  *where V follows N(0,Σ) distribution, and $H=D^{2}f\left(p\right)$ is a Hessian of f.*

In particular, we have the following result for $\hat{JMI}$ [54]:

**Corollary** **1.** *Let Y be binary.*

*(i)*  *If $\sigma_{\hat{JMI}}^{2}\neq 0$ then*
$$n^{1/2}\left(\hat{JMI}-JMI\right)\overset{d}{\to }N(0,\sigma_{\hat{JMI}}^{2}),$$
  *where*
(31) $$\begin{array}{cc} \sigma_{\hat{JMI}}^{2} & =\sum_{x,y,x_{S}}p\left(x,y,x_{S}\right){\left(\frac{1}{\left|S\right|}\sum_{s\in S}\ln\frac{p\left(x,y,x_{s}\right)p\left(x_{s}\right)}{p\left(x,x_{s}\right)p\left(y,x_{s}\right)}\right)}^{2}\\ \; & -{\left(JMI\right)}^{2} \end{array}$$
*(ii)*  *We $\sigma_{\hat{JMI}}^{2}=0\Leftrightarrow JMI=0$. In this case,*
$$2n\hat{JMI}\overset{d}{\to }V^{T}HV=\sum_{i=1}^{M}\lambda_{i}Z_{i}^{2},$$
  *where V and H are defined in Theorem 2, $Z_{i}$ are iid N(0,1) and $\lambda_{i}$ are eigenvalues of the matrix W=ΣH which has the following elements*
$$W_{x,y,x_{S}}^{x^{\prime },y^{\prime },x_{S}^{\prime }}=\frac{1}{\left|S\right|}\sum_{s=1}^{\left|S\right|}\left[\frac{I(x_{s}=x_{s}^{\prime })}{p(x_{s})}-\frac{I(x=x^{\prime },x_{s}=x_{s}^{\prime })}{p(x,x_{s})}-\frac{I(y=y^{\prime },x_{s}=x_{s}^{\prime })}{p(y,x_{s})}+\frac{I(x=x^{\prime },y=y^{\prime },x_{s}=x_{s}^{\prime })}{p(x,y,x_{s})}\right].$$

It follows from Theorem 1 that if for any i∈S we have that $I(Y;X|X_{i})=0$, i.e., *Y* and *X* are conditionally independent given $X_{i}$, then asymptotic distribution is that of quadratic form specified in (ii), otherwise the distribution is normal.

The main advantage of using modified criteria instead of CMI is that their estimation does not require as large samples as for CMI itself. Note that for modifications of order *k*, conditioning involves *k*-dimensional strata, whereas for CMI *p*-dimensional strata are considered. However, modified criteria considered in Section 4.1 suffer from the fact that under hypothesis of conditional independence X⊥⊥Y|Z the asymptotic distribution of empirical criterion is not uniquely determined and has to be estimated from the sample. As in the case of $\hat{JMI}$, the asymptotic distribution can be either normal (when X⫫Y|Xj for at least one j∈S or coincides with distribution of the quadratic form. Which type of asymptotic distribution is valid can be decided using resampling schemes shortly discussed in Section 7. The chosen distribution can used as a benchmark to test the conditional independence hypothesis (see, e.g., [29,55]). Alternatively, testing of $H_{0}:I\left(Y;X|X_{S}\right)=0$ can be replaced by testing ${\tilde{H}}_{0}:I\left(Y;X|X_{i}\right)=0$ for any i∈S at each stage of forward feature selection procedure and the benchmark distribution can be obtained by approximating eigenvalues of *M* specified in (ii) (see [54]) or using scaled and shifted χ squared distribution $\alpha +\beta \chi_{d}^{2}$, where α,β,d are estimated from the sample [56]. Note that, although $H_{0}$ and ${\tilde{H}}_{0}$ are not equivalent, in the case of faithful distributions (See Section 9.2 for definition of faithfulness) ${\tilde{H}}_{0}$ implies $H_{0}$, as conditional independence is inherited by conditioning supersets in such a case.

## 7. Resampling Schemes

When using the conditional independence test, which the test statistic is used for, what is very common, the exact or even the asymptotic distribution under conditional independence is not known, the usual practice is to use conditional randomisation (CR) and resampling schemes to approximate this distribution and use the resulting approximation as the benchmark distribution, as described in the beginning of Section 6. Analogously as before, comparison of the value of the statistic based on the observed sample with the benchmark distribution is used to calculate the CR or resampling *p*-value. It can be also used as alternative way of assessing the distribution of the statistic even when its approximate distribution is known. We briefly discuss these procedures, first for a discrete $X_{S}$, then for a continuous case.

- *Conditional Randomisation* (CR).In the case of the CR approach we assume that the probability mass function $p(x|X_{S}=x_{S})$ or density of *X* given $X_{S}=x_{S}$ is known. Then, given a sample $\left(X,Y,X_{S}\right):={\left(X_{i},Y_{i},X_{S,i}\right)}_{i=1}^{n}$ one generates a CR sample $\left(X^{*},Y,X_{S}\right)={\left(X_{i}^{*},Y_{i},X_{S,i}\right)}_{i=1}^{n}$, when $X_{i}^{*}$ are independently sampled from p.m.f. or density $p(x|X_{S}=x_{S,i})$ for i=1,…,n. Let $T(X,Y,X_{S})$ be a test statistic which we would like to use for testing CI and consider *M* generated CR samples. We define the permutation *p*-value as
  (32) $$p_{CR}=\frac{\#\{k=1,\ldots ,M:T\left(X_{k}^{*},Y,X_{S}\right)\geq T\left(X,Y,X_{S}\right)\}+1}{M+1}.$$
  It follows that $p_{CR}$ is a valid *p*-value, i.e., conditionally on $(\left(Y,X_{S}\right)$, i.e., its distribution is uniform on the set {1/M,2/M,…,M/(M+1)} under CI and, thus,
  $$P(p_{CR}\leq \alpha |Y,Z)\leq \alpha .$$
  This follows from the following simple fact. Suppose that CI holds and $X^{*}$ is such that $P_{X^{*}|X_{S}}=P_{X|X_{S}}$. Then, given $Y,X_{S}$, when CI holds, we have the following equality in distribution
  $$T\left(X,Y,X_{S}\right)\overset{d}{=}T\left(X^{*},Y,X_{S}\right);$$
  (see, e.g., [57]). For recent improvements of the CR method, random permutations are appropriately chosen and considered instead of choosing them at random, see [58]. In the case when $p(x|X_{S}=x_{S})$ is unknown, CR sampling is replaced by Conditional Permutations or Bootstrap X method.
- *Conditional permutation (CP)*. Conditional permutation method is similar to CR method and differs in that for value $x_{S}$ taken by elements of $X_{S}$ we consider the strata of the sample corresponding to this value, namely
  $$P_{i}=\left\{j:\left(X_{j},Y_{j},X_{S,j}\right):X_{S,j}=x_{S,i}\right\}.$$
  The CP sample is obtained from the original sample by replacing $\left(X_{j},Y_{j},X_{S,j}\right)$ for $j\in P_{i}$ by $\left(X_{\pi^{i}\left(j\right)},Y_{j},X_{S,j}\right)$, where $\pi^{i}$ is a randomly chosen permutation of $P_{i}$. Thus, on every strata $X_{S}=x_{s}$ we randomly permute values of corresponding *X* independently of values of *Y*. Once *M* of CP samples are obtained independently in this fashion, we calculate *p*-value $p_{PC}$ based on them analogously as before.
- *Conditional Bootstrap X (CB.X)*. Instead of permuting values of *X* on each strata, we draw a bootstrap sample from *X* observations, that is why we sample them with replacement as many times as is the size of the strata. The remaining steps are as previously described.

We discuss now the resampling for continuous predictors. Feature selection for this case is covered shortly in Section 9. We remark here that the aim, methodology of solutions, and criteria considered are very similar, the main differences consist of technical problems of adequate estimation of information-theoretic measures in the continuous case.

In the case of $X_{S}$ being continuous, CR methods work as stated, however the situation is more complicated for CP and CB.X method as those depend on transforming the strata of the sample, which degenerate to single observation points in this case. One possible solution is to sample from some estimate $\hat{p}\left(x|X_{S}=x_{S}\right)$, e.g., kernel estimate, however this requires a large number of observations on each strata. The following proposal of constructing pseudo sample distributions of which is close to $P_{X,Y,X_{S}}$ under CI has been suggested by [59] and consists in using observations having close values to $X_{S}=x_{S}$. More specifically, consider observation $\left(x_{i},y_{i},x_{S,i}\right)$ and find $k^{th}$ nearest observation to $x_{S,i}$ in $X_{S}$ space, say, $x_{S,j}$ with corresponding triple $\left(x_{j},y_{j},x_{S,j}\right)$. Then, observation of $\left(x_{i},y_{i},x_{S,i}\right)$ is replaced in the resampling sample by $\left(x_{j},y_{i},x_{S,i}\right)$.

Another technique for data augmentation, applicable in both discrete and continuous cases, is *knock-off* construction which we now describe. Consider the regression problem involving vector $X=(X_{1},\ldots ,X_{p})$ of features and response *Y*. Vector $\tilde{X}=\left({\tilde{X}}_{1},\ldots ,{\tilde{X}}_{p}\right)$ is the knock-off vector for *X* in this regression problem, if $Y⊥\;\;\;⊥\tilde{X}$ and, moreover, for any S⊆{1,…,p} we have the following equality in distribution [57]:

$${\left(X_{1},\ldots ,X_{p},{\tilde{X}}_{1},\ldots ,{\tilde{X}}_{p}\right)}_{swap(S)}\overset{d}{=}\left(X_{1},\ldots ,X_{p},{\tilde{X}}_{1},\ldots ,{\tilde{X}}_{p}\right)$$

where swap(S) means swapping entries $X_{j}$ and ${\tilde{X}}_{j}$ for any j∈S. Thus, in the above sense ${\tilde{X}}_{1},\ldots ,{\tilde{X}}_{p}$ are interchangeable with $X_{1},\ldots ,X_{p}$ and independent of *Y* at the same time.

The construction of knock-offs is complicated in general, feasible only in special cases as of now, such as the Gaussian case, and requires knowledge of distribution of $P_{X}$. Thus, even more information is needed than in the case of CR approach. However, the gains are considerable, as for natural classes of statistics measuring influence of predictors on the response one can compare the performance of $X_{1},\ldots ,X_{p}$ with that of their knock-offs and on this basis decide which ones are not strongly relevant. Intuitively, when the performance of $X_{j}$ measured, e.g., by an absolute value of estimated regression coefficient, is comparable to that of its knock-off, then such a variable should be discarded. This approach yields a bound on FDR of strongly relevant features (Theorem 3 in [26]). Thus, in cases discussed in Section 3, when the set of strongly relevant features is exactly equal MB(Y), this approach yields a bound on FDR of its recovery. We stress that, in this way, one can analyse properties of the whole procedure of MB determination, and not only that of an individual steps in the algorithm. An additional advantage is that, unlike for the resampling schemes above, only one sample of knock-offs needs to be generated.

## 8. Markov Blanket Discovery Algorithms

We discuss now Markov Blanket discovery algorithms. Their aim is to solve a feature selection problem posed in Section 3 (see Equation (11)) applying CMI or CMI approximations introduced in Section 4 for which theoretical guarantees are discussed in Section 5. Such algorithms are used as building blocks for conditional independence tests, based either on asymptotic distributions of test statistics discussed in Section 6 or approximations of their distributions based on resampling covered in Section 7.

We discuss three representative examples of Markov Blanket discovery algorithms. Other examples include [60] and algorithms using assumed faithful Directed Acyclic Graph (DAG) representation of the underlying probability structure (for DAG fathfulness of probability distribution based on *d*-separation (see e.g., [4]), such as HITON-MB [61], MMMB [62], IPC-MB [63], and STMB [64].

- The GS (Grow and Shrink) algorithm [65]. It consists of two phases. Specific ordering of variables in *F* is considered, e.g., variables may be ordered according to value of I(Y;X) and, then, the variable having the largest value of the mutual information is the first variable chosen. Denoting by *S* the current set of chosen variables, we pick the first variable (in the considered ordering) which depends on *Y* given $X_{S}$ ($I(Y;X|X_{S})>0$) and we add it to *S*, then repeat the step. When there is no longer such a variable among candidates, the first phase is terminated. We call the resulting chosen set $S^{*}$. In the second phase, we remove from $S^{*}$, again using the considered ordering, any variable $X_{j}$ which is not strongly relevant with respect to the current $S^{*}$, i.e., such that $X_{j}⊥\;\;\;⊥Y|X_{S^{*}\backslash \left\{j\right\}}$ ($I(Y;X_{j}|X_{S^{*}\backslash \left\{j\right\}})=0)$ and we let $S^{*}:=S^{*}\backslash \left\{j\right\}$.
- The IAMB (Incremental Association Markov Blanket) [66]. The algorithm is similar to GS with one important difference in the first step. Namely, it disregards initial ordering and *S* is augmented by the most plausible candidate, that is the variable realising $\max_{i\in S^{c}}I\left(X_{i},Y|X_{S}\right)$, provided it is not conditionally independent from *Y* given $X_{S}.$
- $GS^{\left(m\right)}$ ([27], where m≤p. $GS^{\left(m\right)}$ differs from GS in the growing phase only. Namely, instead of an *individual* variables with indices in F\S being considered as possible candidates, all *subsets T* of size not exceeding *m* are taken into account and the check is performed whether there are conditionally dependent on *Y* given current *S*. If this holds S:=S∪T.

It is proved in [27] that $GS^{\left(m\right)}$ algorithm yields the *m*-Markov Blanket (see Section 3 for definition of *m*-Markov Blanket) of *Y*. Thus, for m=p we have that the output of $GS^{\left(p\right)}$ is MB(Y). However, since, in the growing phase, all subsets of F\S of size not exceeding *m* have to be checked which is computationally intensive, in practice *k* subsets for *k* large enough are checked at every step.

Note that for such results we assume that condition $Y⊥\;\;\;⊥X_{T\backslash S}|X_{S}$ or $Y⊥\;\;\;⊥X|X_{S}$ can be verified. In practice, it is impossible to check conditional independence of *X* and *Y* given the set of variables already chosen without error and this has to be replaced with the appropriate test discussed in Section 6. Obviously, as such a test has to be performed at each step, one does not control probability of including false discoveries. False discoveries are dealt with in the second phase, but no formal results exist concerning how likely recovering MB(Y) is in the case of practical algorithm. However, see [67] for the results concerning the PC algorithm in the Gaussian case and knock-off construction discussed in Section 7.

Another possibility is to omit phase two and stop early phase one, applying stopping rules devised in multiple testing problems to control Family-Wise Error Rate or False Discovery Rate (see, e.g., [68] where a stopping rule using the Holm procedure has been applied for CIFE criterion). These methods work promisingly in practice, however, due to the fact that the set *S* augmented at each step is data-dependent, also in this case formal results on the consistency of such procedures are not available.

There is no definitive study of empirical performance of the presented criteria and/or selection procedures; note that any criterion considered can be used for any feature selection method described above, thus creating a large number of filter methods. Moreover, those can be evaluated using their classification performance, as well as for synthetic datasets, according to their ability to choose active predictors. Thus, we discuss only the reports on the simplest greedy procedures consisting on the application of discussed criteria on synthetic datasets with a fixed ahead number of chosen features. Authors of [24] discuss, among other things, results for datasets coming from NIPS Feature Selection Data Challange, Gisette, and Madelon (procedures stopped at 200 variables in the first case and 20 in the second). Two interesting points arise from the study: strong performance of JMI, which was the second best and co-winner in those cases, with respect to balanced accuracy (BA) and the number of feature chosen, with a strong performance of CMIM (winner in the case of Gisette and together with CIFE co-winner in the case of Madelon). The overall strong performance of CIFE was also confirmed in [68,69]. The second important observation is the failure of performance of CMI, which due to scarcity of data, failed to detect conditionally dependent variables very early. This is due to the fact that for a large conditioning set the test becomes very conservative (see also [70] for discussion of this property).

## 9. Case of Continuous Distribution $P_{X,Y,Z}$

In the following, we shortly discuss conditional independence testing for continuous distributions. The main motivation to include a continuous case in this review, devoted mainly to discrete case, is to underline the strong similarities between these two cases. In particular, we discuss below that all information-theoretic tools defined for the discrete case have their analogues for continuous distributions. Moreover, we note that the selection methods presented till now do not depend on continuity of the underlying measure, once a specific test for conditional independence X⊥⊥Y|Z which takes this into account is used. Thus, the differences between two cases are mostly due to the fact that estimation of information-theoretic measures are much more difficult for continuous distributions. Moreover, their asymptotic behaviour under CI is not known, thus making construction of corresponding tests difficult. On the other hand, for Gaussian case significant simplifications exists due to the existence of closed formula for CMI discussed in Section 9.2.

### 9.1. General Considerations

Returning to notation of Section 2, we discuss conditional independent testing in a continuous case. In this context, it is enlightening to mention the result in [71], where it is shown that in the continuous case conditional independence testing is a hard problem in the following sense. For any test on a natural family of $P_{X,Y,Z}$ defined in [71] satisfying X⊥⊥Y|Z, which *uniformly* achieves type I error asymptotically not larger than a prescribed significance level α its power for *any* alternative will be also no larger than α. Thus, such a test is useless for discriminating between CI and Conditional Dependence.

The indices defined in Section 2 have their natural analogues in the continuous case; namely probabilities P(X=x,Y=y,Z=z) are replaced by density functions p(x,y,z) and summations by integrals (for details see [15]). What is important for development here is that Conditional Independence X⊥⊥Y|Z in the continuous case is also equivalent to conditional Mutual Information I(X;Y|Z) being 0, as in the discrete case. Thus, the problem boils down to an accurate estimation of I(X;Y|Z) and corresponding testing of CI using constructed estimator as a test statistic. This, however, is also a much harder task than in a discrete case as plug-in estimators of entropy, and conditional and unconditional information when, e.g., kernel estimators of densities (see, e.g., [6], Chapter 6) are plugged-in are unstable and work satisfactorily only when very large samples are available. Much better results are obtained using the variational approach described in Section 4.2. Additionally, some estimators based on the nearest neighbour idea, such as the Kozachenko–Leonenko estimator of entropy [72,73] and its refinements, such as the Kraskov et al. estimator [74] work better than straightforward plug-in estimator. Then, estimators of MI are constructed using (1). Additionally, the simple approach based on discretising underlying variables is frequently used. There are two problems related to this approach. The first one is a loss of information due to this operation. The second, related one, is a difficulty of choosing an appropriate bin size, especially in many dimensions. Too large bin size may result in hiding interesting characteristics of predictors’ distributions and, consequently, a loss of predictive power.

We mention two other approaches for the continuous case. One which is frequently used is *kernel based approach* which relies on the following fact [75]:

$$X⊥\;\;\;⊥Y|Z\Leftrightarrow Ef\left(X,Z\right)h\left(Y,Z\right)=0\mathrm{for}\mathrm{any}f\in F_{X|Z},h\in F_{Y|Z},$$

where $F_{X|Z}=\left\{h\left(Y,Z\right):h\left(Y,Z\right)=h_{0}\left(Y\right)-E\left(h_{0}\left(Y\right)|Z\right),h_{0}\in L_{Y}^{2}\right\}$. Thus, although conditional independence is not equivalent to conditional covariance being zero, this is true when transformations of vectors (X,Z) and (Y,Z) are allowed. In view of this result it turns out that its possible to find rich enough function spaces, such that the condition that the generalized conditional covariance of transformed *X* and *Y* being equal to 0 is equivalent to conditional independence. Moreover, it is the function space that one can consider to appropriately define Reproducing Kernel Hilbert Spaces (RKHS). This, conceptually, is much more involved than checking I(X;Y|Z) being 0, save for the technical problems of testing this condition in the continuous case.

We also mention in this context the second approach, called distillation method [76] which relies on ideas similar to the construction of partial residual plot and resampling.

### 9.2. Gaussian Case

We review the case when (Y,X) is Gaussian, where $X=(X_{1},\ldots ,X_{p})$. This case offers significant simplifications, as the quantities on which feature selection is based can be explicitly calculated. Namely, direct calculation yields [15]:

$$H\left(X\right)=\frac{1}{2}\log\left|\Sigma_{X}\right|+\frac{p}{2}\log\left(2\pi e\right),$$

if $X∼N(\mu_{X},\Sigma_{X})$. If $\left(X,Y\right)∼N\left(\mu_{X,Y},\Sigma_{X,Y}\right)$ it follows from (2) that

$$I\left(X;Y\right)=\frac{1}{2}\log\frac{|\Sigma_{X,Y}|}{|\Sigma_{X}\left|\times \right|\Sigma_{Y}|},$$

where |Σ| stands for determinant of matrix Σ. In particular for bivariate normal case when ρ(X,Y)=ρ we have $I\left(Y;X_{1}\right)=-\left(\log\left(1-\rho^{2}\right)\right)/2$. Whence, in the case of the conditional distribution of (*Y*, *X*), given $X_{S}$, where $X_{S}\subseteq \left\{2,\ldots ,p\right\}$ this yields

$$I\left(Y;X_{1}|X_{S}\right)=-\frac{1}{2}\log\left(1-\rho_{par}^{2}\right),$$

where $\rho_{par}$ is partial correlation coefficient between *Y* and $X_{1}$ given $X_{S}$. Thus, in Gaussian case information-based dependence indices can be expressed in terms of either correlation or partial correlation coefficients.

In order to present the PC algorithm, we first introduce the concept of the faithfulness of the distribution which plays an important role in its construction (see, e.g., [67]). Let $X_{0}:=Y$ and consider undirected graph G=(V,E), where vertices V={0,1,…,p} correspond to variables $\left(Y,X_{1},\ldots ,X_{p}\right)$ and E⊂V×V, such that (j,k)∈E⇔(k,j)∈E is the set of edges.

**Definition** **8.** *Distribution $P_{Y,X}$ is faithful to graph G when for any triple A,B,C⊂V we have*

(33) $$A\;\mathrm{and}\;B\;\mathrm{are}\;\mathrm{separated}\;\mathrm{by}\;C\;\Leftrightarrow \;X_{A}⊥\;\;\;⊥X_{B}|X_{C},$$

*when separation by C means that every path joining elements of A and B has to pass through C. (Implication from LHS to RHS in (33) is called global Markov property.)*

In the case when $\left(Y,X_{1},\ldots ,X_{p}\right)$ is Gaussian and there exists faithful representation of its distribution, PC algorithm [77] reconstructs its dependence structure with probabilistic guarantees. The main tool is clever usage of one of the consequences of faithfulness, namely

(34) $$X_{1}⊥\;\;\;⊥X_{2}\left|X_{C_{1}}\;\Rightarrow X_{1}⊥\;\;\;⊥X_{2}\right|X_{C_{2}}\;\;\mathrm{for}\mathrm{any}\;C_{2}⊇C_{1}.$$

The algorithm starts from the fully connected graph and removes edges between any vertices which are marginally uncorrelated (and, thus, in view of (34) and Gaussianity, conditionally independent given any subset of variables). Then, it proceeds to remove edges between vertices which correspond to variables which are conditionally independent given a subset of their neighbours of size *l*, where l=1,2,… is increased stepwise. The conditional independence is tested using an empirical partial correlation coefficient ${\hat{\rho }}_{par}$ and the property that its Fisher transform $log[\left(1+{\hat{\rho }}_{par}\right)/\left(1-{\hat{\rho }}_{par}\right)]$ is approximately N(0,1) normal under CI (details in [67], Section 13.7).

The case when *Y* is discrete and $P_{X|Y=y_{i}}$ are normal is more complicated, as in this scenario distribution of *X* is that of normal mixture for which no explicit formulae for its entropy exist (see, however, [34] for special cases of mixtures of summands having the same covariance matrix). Authors of [78] use approximations of MI (see (18) in [78]) to address this problem.

To summarise, we note that the section discusses possible approaches to test conditional independence and discover Markov Blanket of target *Y* when the underlying distribution is continuous. The first task can be performed by using some available estimators of CMI, discussed above, such as Kraskov et al. estimator [74] in conjunction with benchmark distribution based on resampling. The other possibility is a kernel method which reduces the problem to checking whether generalised conditional covariance is 0. For the parametric case when (X,Y) is Gaussian, the testing problem can be reduced to testing whether the partial correlation coefficient is 0. Moreover, in this case under faithfulness assumption on the distribution Markov Blanket can be recovered with theoretical guarantees.

## 10. Interaction Detection

Detection of existing interactions between features in influencing the outcome *Y* is an important task of data analysis; primary examples being gene–gene interactions in Genome-Wide Association Studies (GWAS) and gene–environment interactions. In this section, we show how information-theoretic measures introduced before can be applied to solve this problem. Moreover, we indicate that interaction detection problems may be viewed as a special case of feature selection. Various indices have been proposed to measure the strength of interactions, one of the most popular tools being interaction information discussed in Section 2 and Section 4.1. There it was considered as a tool to define new selection criteria by truncating and possibly weighing summands in the Möbius expansion of CMI. Here, II is adopted as a main tool in detection of interactions. Its most popular competitor is the value of Likelihood Ratio Test statistics for two nested logistic models: an additive model with no interactions and a saturated model taking all possible interactions into account. However, it has been shown in [68] that for logistic model when predictors are independent and at least one of interaction terms is non-zero then II≠0 but not vice versa. This shows that there are situations when interactions are present and are detected by II but will go undetected using logistic model approach. II is applied as the main tool to find interactions in AMBIENCE package [79] and BOOST package uses so called Kirkwood approximation discussed below, which is closely related to II [80]. Other competitors to LRT and II include Multifactor Dimensionality Reduction (MDR) [81] and Restricted Partitioning Method (RPM) [82].

Now, we will discuss two additional properties of Interaction Information which are useful in interaction analysis, focusing on its three-way variant applied to predict synergistic effect of two variables $X_{1}$ and $X_{2}$, say, in determining the target *Y*.

From the first equality in (5) it follows that when both variables are jointly independent from *Y*, i.e., $(X_{1},X_{2})⊥\;\;\;⊥Y$ then $II(X;X_{2};Y)=0$ as $I\left(X_{1};X_{2}|Y\right)=I\left(X_{1};X_{2}\right)$. Although the converse is not true, it can be shown in adversarially constructed examples only and, thus, testing $H_{0}:II=0$ is usually replaced by (more stringent) hypothesis ${\tilde{H}}_{0}:\left(X_{1},X_{2}\right)⊥\;\;\;⊥Y$.

The other property is insightful representation

$$II\left(X_{1};X_{2};Y\right)=KL(P_{X_{1},X_{2},Y}\left|\right|{\tilde{P}}_{K}),$$

where ${\tilde{P}}_{K}$ is Kirkwood Superposition Approximation with masses assigned to points equal

$${\tilde{p}}_{K}\left(x_{1},x_{2},y\right)=\frac{p\left(x_{1},x_{2}\right)p\left(x_{1},y\right)p\left(x_{2},y\right)}{p\left(x_{1}\right)p\left(x_{2}\right)p\left(y\right)}$$

is positive but necessarily summing up to 1, mass function supported on values $\left(x_{1},x_{2},y\right)$ of $\left(X_{1},X_{2},Y\right)$. It can be shown that for η denoting the summary mass of ${\tilde{P}}_{K}$ it follows that when η≤1 and II=0, then $P_{X_{1},X_{2},Y}$ is equal to its Kirkwood approximation [68].

Let us discuss two commonly used methods of testing that $II(X;X_{2};Y)=0$. The most popular one is based on Han’s approximation [20] derived under a stringent assumption of overall independence, which results in considering as the benchmark distribution chi-squared distribution with (I−1)(J−1)(K−1) degrees of freedom, when I,J,K are numbers of values of $X_{1},X_{2}$, and *Y*, respectively. This, however, may lead to a large number of false signals when the pertaining test is employed as the overall independence is only a very special situation when II vanishes. It is shown in [69] that when $\left(X_{1},X_{2}\right)$ are independent of *Y*, similarly to other measures of conditional dependence discussed in Section 4.1, the approximate distribution is weighted chi-square distribution. Its complete description has been given for $X_{1}$ and $X_{2}$ having three values and *Y* being binary, which includes typical GWAS situation of two loci with two co-dominant alleles. The properties of the corresponding test have not been investigated yet.

The other method uses permuting *Y* values of the considered sample and in this way obtaining *M* random samples satisfying ${\tilde{H}}_{0}:\left(X_{1},X_{2}\right)⊥\;\;\;⊥Y$. Then, permutation *p*-value can be calculated as ${\left(M+1\right)}^{-1}\left(1+\sum_{i=1}^{M}I\left({\hat{II}}_{i}\geq \hat{II}\right)\right)$ (compare (32)). One can also use chi-square distribution with the number of degrees of freedom as the benchmark distribution, analogously to [70]. The advantage of the latter approach is that the number of permuted samples *M* may be much smaller than in the case of permutation test.

The main challenge to detect significant interactions among potential predictors F={1,…,p} is usually computational burden of the procedure. Indeed, any method discussed should in principle include interaction term for any pair of predictors $X_{i},X_{j},i,j\in F$. This requires enlarging set *F* by |F|×(|F|−1)/2 hot-encoded interactions, i.e., fitted model will contain |F|×(|F|+1)/2 nominal predictors. This is practically infeasible, e.g., for 100 Single Nucleotide Polymorphisms (SNP) being our predictors this would yield number of needed tests of order $10^{6}$ at each step of selection procedure. The frequently adapted approach is to screen the set of variables first and then apply more sophisticated procedure to the chosen variables only.

Consider two other possible solutions. The first one uses the premise that significant interactions may arise only among variables which themselves are significant. Thus any of the method described in Section 8 can be used for predictors in *F* and then two methods described above are applicable for all interactions of the chosen features using Bonferroni adjustment for multiple testing or Holm method [83]. The problematic aspect of this approach is exclusion of possible interactions between two features when only one of them has non-negligible main effect.

The other group of methods, for which BOOST proposed in [80] is a representative example, screens interactions by the modified version of LRT test which consists of replacing likelihood for a fitted additive model by likelihood for normalised Kirkwood approximation ${\tilde{P}}_{K}/\eta$ which can be very quickly computed. The pairs for which the value of LRT statistic modified in this way exceeds certain threshold. Then, an exact LRT test is performed for all remaining pairs. The weak side of this approach is a choice of a threshold τ in the first stage which needs to be chosen by a rule-of-thumb as properties of modified LRT test remain unknown.

## 11. Conclusions

This paper reviews main ideas concerning feature selection using information theoretic tools which revolve around detecting and measuring the strength of conditional independence. As estimation of CMI, which is a natural and powerful tool for this endeavour, encounters a problem when dimensionality of the task is large; a natural way is to look for its good substitutes. Several ways in which such substitutes are constructed are discussed and the current knowledge about their properties is presented. It is argued that selection criteria based on truncation of Möbius decomposition make quite far-reaching compromises on the dependence structures of feature sets, whereas properties of approximations based on variational approaches are yet to be established. Additionally, major Markov Blanket discovery algorithms are constructed under assumptions that conditional independence or its absence can be established without error, when, in reality, the intrinsic features of any practical CI test applied at each stage is its fallibility. Therefore, further efforts, both theoretical and experimental, are needed to understand the advantages, drawbacks, and interrelations between up-to-date developments. The paper presents some recently established tools which can be used for this purpose, such as asymptotic distributions of CMI-related criteria discussed in Section 6. They are used to construct tests of conditional independence with approximate control of probability of false signals. The problem of existence of interactions can be similarly approached using results in [69].

Another challenging task is to extend general approaches discussed here, such as construction of feature selection criteria by truncation of Möbius expansion or variational approach to multilabel classification when *Y* is a multivariate binary vector. Several criteria, such as CIFE or JMI, have been generalised to this case already (cf. [84,85], respectively, see also [86] for an approach based on Han’s inequality and [87] for the review), but the general approach, e.g., in the spirit of [24], is still missing. Another important challenge here seems construction of feature selection criteria which will efficiently take into account dependence between coordinates of the response.

## Data Availability

Not applicable.

## Appendix A

Proof of Theorem 3.

**Proof.** (i) follows from the first equality in (16) after noting that the naive Bayes assumption implies in particular that: $X_{S}⊥\;\;\;⊥X|Y$ and, thus $I(X;X_{S}|Y)=0$. Moreover we have

$$I\left(X;X_{S}\right)=H\left(X_{S}\right)-\sum_{i\in S}H\left(X_{i}|X\right)=H\left(X_{S}\right)-\sum_{i\in S}H\left(X_{i}\right)+\sum_{i\in S}I\left(X;X_{i}\right).$$

The conclusion follows as two first terms do not depend on *X*. (ii) follows directly from Möbius formula and in order to prove (iii) using I(X;Y)=H(Y)−H(Y|X) we have

(A1) $$\begin{array}{ccc} I(Y;X|X_{S}) & = & I\left(X;Y\right)-I\left(X;X_{S}\right)+I\left(X;X_{S}|Y\right)\\ & = & I\left(X;Y\right)-H\left(X_{S}\right)+H\left(X_{S}|X\right)+H\left(X_{S}|Y\right)-H\left(X_{S}|X,Y\right) \end{array}$$

and, thus, up to terms which do not depend on *X* it equals in view of assumptions

$$\begin{array}{ccc} & & I\left(X;Y\right)+H\left(X_{S}|X\right)-H\left(X_{S}|X,Y\right)=I\left(X;Y\right)+\sum_{i\in S}\left(H\left(X_{i}|X\right)-H\left(X_{i}|X,Y\right)\right)=\\ & & I\left(X;Y\right)+\sum_{i\in S}I\left(X_{i};Y|X\right), \end{array}$$

which differs from $CIFE(Y,X|X_{S})$ by yet another term which does not depend on *X*. (iv) We show now reasoning leading to Joint Mutual Information Criterion JMI (cf. [33,48]). Namely, we have for i∈S

$$I\left(X;X_{S}\right)=I\left(X;X_{i}\right)+I\left(X;X_{S\backslash \{i\}}|X_{i}\right).$$

Summing these equalities over all i∈S and dividing by |S| we obtain

$$I\left(X;X_{S}\right)=\frac{1}{\left|S\right|}\sum_{i\in S}I\left(X;X_{i}\right)+\frac{1}{\left|S\right|}\sum_{i\in S}I\left(X;X_{S\backslash \{i\}}|X_{i}\right)$$

and analogously

$$I\left(X;X_{S}|Y\right)=\frac{1}{\left|S\right|}\sum_{i\in S}I\left(X;X_{i}|Y\right)+\frac{1}{\left|S\right|}\sum_{i\in S}I\left(X;X_{S\backslash \{i\}}|X_{i},Y\right).$$

Subtracting two last equations and using definition of II we obtain

$$I\left(Y;X|X_{S}\right)=I\left(X;Y\right)+\frac{1}{\left|S\right|}\sum_{i\in S}II\left(X;X_{i};Y\right)+\frac{1}{\left|S\right|}\sum_{i\in S}II\left(X;X_{S\backslash \left\{i\right\}};Y|X_{i}\right).$$

Moreover it follows from definition of II that when *X* is independent from $X_{S\backslash \{i\}}$ given $X_{i}$ and these quantities are independent given $X_{i}$ and *Y* the last sum is 0 and we obtain definition of JMI. □
